# Bacteriocin Producing *Streptococcus agalactiae* Strains Isolated from Bovine Mastitis in Brazil

**DOI:** 10.3390/microorganisms10030588

**Published:** 2022-03-09

**Authors:** João Ricardo Vidal Amaral, Rommel Thiago Jucá Ramos, Fabrício Almeida Araújo, Rodrigo Bentes Kato, Flávia Figueira Aburjaile, Siomar de Castro Soares, Aristóteles Góes-Neto, Mateus Matiuzzi da Costa, Vasco Azevedo, Bertram Brenig, Selma Soares de Oliveira, Alexandre Soares Rosado

**Affiliations:** 1 Institute of Microbiology, Universidade Federal do Rio de Janeiro, Cidade Universitária, Rio de Janeiro 21941-902, RJ, Brazil; joaoricardo.vidal@gmail.com (J.R.V.A.); selma@micro.ufrj.br (S.S.d.O.); 2 Institute of Biological Sciences, Universidade Federal do Pará, Belém 66075-110, PA, Brazil; rommelthiago@gmail.com; 3 Socio-Environmental and Water Resources Institute, Universidade Federal Rural da Amazônia, Belém 66077-830, PA, Brazil; araujopa@gmail.com; 4 Institute of Biological Sciences, Universidade Federal de Minas Gerais, Belo Horizonte 31270-901, MG, Brazil; rbkato@gmail.com (R.B.K.); faburjaile@gmail.com (F.F.A.); arigoesneto@gmail.com (A.G.-N.); vascoariston@gmail.com (V.A.); 5 Institute of Biological and Natural Sciences, Universidade Federal do Triângulo Mineiro, Uberaba 38025-180, MG, Brazil; siomars@gmail.com; 6 Department of Biological Sciences, Universidade Federal do Vale do São Francisco, Petrolina 56304-917, PE, Brazil; mateus.matiuzzi@gmail.com; 7 Department of Molecular Biology of Livestock, Institute of Veterinary Medicine, Georg August University Göttingen, 37077 Göttingen, Germany; bbrenig@gwdg.de; 8 Biological and Environmental Science and Engineering Division, King Abdullah University of Science and Technology, Thuwal, Makkah 23955, Saudi Arabia

**Keywords:** antimicrobial peptides, antimicrobial resistance, genomics, *Streptococcus agalactiae*

## Abstract

Antibiotic resistance is one of the biggest health challenges of our time. We are now facing a post-antibiotic era in which microbial infections, currently treatable, could become fatal. In this scenario, antimicrobial peptides such as bacteriocins represent an alternative solution to traditional antibiotics because they are produced by many organisms and can inhibit bacteria, fungi, and/or viruses. Herein, we assessed the antimicrobial activity and biotechnological potential of 54 *Streptococcus agalactiae* strains isolated from bovine mastitis. Deferred plate antagonism assays revealed an inhibition spectrum focused on species of the genus *Streptococcus*—namely, *S. pyogenes*, *S. agalactiae*, *S. porcinus*, and *S. uberis*. Three genomes were successfully sequenced, allowing for their taxonomic confirmation via a multilocus sequence analysis (MLSA). Virulence potential and antibiotic resistance assessments showed that strain LGMAI_St_08 is slightly more pathogenic than the others. Moreover, the *mreA* gene was identified in the three strains. This gene is associated with resistance against erythromycin, azithromycin, and spiramycin. Assessments for secondary metabolites and antimicrobial peptides detected the bacteriocin zoocin A. Finally, comparative genomics evidenced high similarity among the genomes, with more significant similarity between the LGMAI_St_11 and LGMAI_St_14 strains. Thus, the current study shows promising antimicrobial and biotechnological potential for the *Streptococcus agalactiae* strains.

## 1. Introduction

In the past few decades, antibiotics have played an essential role in treating infectious diseases and in antimicrobial prophylaxis for surgical procedures [1]. However, that has not always been the case in history, and microorganisms’ infections used to be partially responsible for a lower global average life expectancy. In 1928, Alexander Fleming discovered the first antibiotic, a compound later named as penicillin, which revolutionized treatments of infectious diseases. After its market introduction in the 1940s, mortality rates due to syphilis were drastically reduced. Penicillin’s success created an appeal for the discovery of other similar substances. The collective effort to find new antibiotics resulted in the discovery of several other antimicrobial drugs during the antibiotic golden age, such as sulphonamides and streptomycin for treating scarlet fever and tuberculosis, respectively [2].

Unfortunately, most of the original antibiotics and some of their later modifications have been ineffective in treating infectious diseases in recent years. Antibiotic resistance is a natural process in which microorganisms find ways to circumvent the action of drugs designed to kill them [1]. The accelerated selection of resistant strains occurs due to the widely adopted use of antibiotics in humans, animals, and crops [3]. The discovery pace of new classes of antibiotics has slowed ever since the antibiotic golden age to top it off [2]. Therefore, antibiotic resistance is one of the most significant global health challenges of our time. Statistical analyses predict that 10 million people are expected to die every year due to untreated microbial infections by 2050 if antimicrobial resistance continues to rise [4].

*Streptococcus agalactiae* (also known as group B *Streptococcus*) is an opportunistic pathogen that colonizes the human urogenital and digestive tracts and is associated with life-threatening complications for invasive infections in pregnant women [5,6]. This bacterial species is also responsible for mastitis infections in cattle and economic loss in dairy farms [7]. Penicillin is the antibiotic used to treat *Streptococcus agalactiae* infections. However, in cases of penicillin allergy, clindamycin and erythromycin are used as second-line antibiotics. Reports on clinical isolates with resistance to erythromycin and clindamycin are increasingly frequent [5,6], which highlights the need for the monitoring of antibiotic resistance and research on alternative antimicrobial substances for treating these infections.

There are many alternative methods available and under consideration for solving this problem. One approach to solving this crisis involves using antimicrobial peptides [1]. Antimicrobial peptides are small molecules of proteinaceous nature produced by all organisms that help protect them from infections by microorganisms they may encounter [8]. The last antimicrobial peptide approved for use was daptomycin, isolated from soil bacteria *Streptomyces roseosporus* in 1986. Nonetheless, many antimicrobial peptides are currently under different stages of clinical trials and are expected to be applied in healthcare in the future. Among the 36 antimicrobial peptides under clinical trial in 2019, 5 were of bacterial origin. It is worth mentioning that most of the antimicrobial peptides under clinical trials are of human origin due to their expected low toxicity. However, bacterial antimicrobial peptides are not to be ignored, especially bacteriocins, since they provide a vast diversity of substances with targeted action [9]. Bacteriocins are a class of ribosomally synthesized antimicrobial peptides produced by bacteria and are biologically active against other Gram-positive and Gram-negative bacteria [10].

Lactic acid bacteria represent a group of Gram-positive bacteria, which are important due to their production of lactic acid, organic acids, secondary metabolites, and their potential application as probiotics [11]. This group comprises several genera—namely, *Aerococcus*, *Carnobacterium*, *Enterococcus*, *Lactobacillus*, *Lactococcus*, *Leuconostoc*, *Oenococcus*, *Pediococcus*, *Streptococcus*, *Tetragenococcus*, and *Weisella* [12]. Many bacteriocins have been isolated from this group and are used in fermentation processes in the food industry [13].

More specifically for the genus *Streptococcus*, bacteriocins have been described from some species, such as *Streptococcus bovis* (Bovicin HJ50) [14], *Streptococcus mutans* (Mutacins B-Ny266, II, 1140/III) [15,16,17], *Streptococcus pyogenes* (Streptococcins A-FF22, A-M49 and A-M57, Streptin, Salivaricin A, SpbMN, Blp1, and Blp2) [18], *Streptococcus salivarius* (Salivaricin-A sa, Salivaricin A3, Salivaricin 9) [19,20,21], *Streptococcus thermophilus* (Thermophilin 347) [22], and *Streptococcus uberis* (Ubericin A, and Uberolysin) [23,24]. Furthermore, the Agalacticin has been recently identified in *Streptococcus agalactiae* ATCC 13813 [18]. Furthermore, the production of bacteriocin-like substances in a *Streptococcus agalactiae* strain isolated from vaginitis in Iraq was also reported [25].

Considering the antibiotic resistance crisis as a One Health problem, as it involves humans, animals, and the environment, it is of particular ongoing interest to apply an omics bioinformatics approach for the discovery of new antimicrobial drugs and the monitoring of antimicrobial resistance spread [10]. Therefore, we aimed to evaluate the biotechnological potential of *Streptococcus agalactiae* strains on their production of antimicrobial substances, focusing on the inhibition of pathogenic bacteria. Since the *Streptococcus* genus has a diversity of bacteriocins described, but there is still a lack of knowledge for the *Streptococcus agalactiae* species, we also aimed to sequence a few selected *Streptococcus agalactiae* genomes and apply a genomics approach to assess the presence of gene clusters for antimicrobial substances and secondary metabolites, as well as to monitor antibiotic resistance in these strains.

## 2. Materials and Methods

### 2.1. Strains, Media, and Growth Conditions

In this study, 54 *Streptococcus agalactiae* strains isolated from bovine mastitis were tested for their ability to produce antimicrobial substances. These strains were isolated from dairy farms in the states of São Paulo and Minas Gerais in Brazil (Table 1) and phenotypically identified via biochemical tests in a previous study [26].

Seventy-five strains were used as indicator bacteria in the deferred growth inhibition assays, accounting for *Listeria innocua* (*n* = 1), *Micrococcus* sp. (*n* = 1), *Lactococcus lactis* (*n* = 1), *Cellulomonas fimi* (*n* = 2), *Klebsiella pneumoniae* (*n* = 2), *Staphylococcus aureus* (*n* = 5), *Streptococcus uberis* (*n* = 12), *Streptococcus porcinus* (*n* = 12), *Streptococcus agalactiae* (*n* = 17), and *Streptococcus pyogenes* (*n* = 20). *Klebsiella pneumoniae* KPC strain shows resistance to carbapenems, and *Staphylococcus aureus* ATCC 33591 shows resistance to methicillin. *Micrococcus* sp. and *Cellulomonas fimi* strains were used due to their known susceptibility to diverse antimicrobials. Information on antibiotic resistance to the other indicator bacteria was not determined.

*Streptococcus* spp., *Listeria innocua*, and *Lactococcus lactis* were grown on Brain and Heart Infusion media. *Staphylococcus aureus* and *Klebsiella pneumoniae* were grown in TSB media. *Micrococcus* sp. and *Cellulomonas fimi* were grown on LB media. The antagonism tests were conducted on BHI media, and all bacteria were incubated at 37 °C 18 h before experiments.

### 2.2. Deferred Growth Inhibition Assays

Deferred growth inhibition assays were performed to test the 54 *Streptococcus agalactiae* strains for their ability to produce antimicrobial substances. The inhibition assays were performed following the spot-on-lawn method, as previously described in [27]. For this, *Streptococcus agalactiae* strains were inoculated in 4 mL of BHI broth and incubated at 37 °C overnight. From those cultures, 10 µL was collected and inoculated on sterile BHI plates in the form of circular spots. Then, the plates were incubated at 37 °C overnight. After that, the plates were exposed to cotton balls soaked in 1 mL of chloroform for 30 min to kill the bacteria. After treatment with chloroform vapor, indicator strains were inoculated in 4 mL of BHI semisolid medium and poured on top of the plates, which were incubated at 37 °C overnight. The absence of growth of the indicator strains on top of the *Streptococcus agalactiae* spots was considered a positive result of inhibition. In the presence of a clear zone of inhibition surrounding the *Streptococcus agalactiae* spots, the size of the inhibition zones was measured in millimeters. The sterile BHI medium was used as the negative control.

The *Streptococcus agalactiae* strains were first tested against *Listeria innocua*, *Streptococcus pyogenes*, *Streptococcus agalactiae* I14, *Cellulomonas fimi* NCTC 7547, *Lactococcus lactis*, and *Staphylococcus aureus* RN451.

Additional tests were carried out with the LGMAI (Laboratory of Genetics of Microorganisms associated with Food and Industry) bacteriocin producing strains 3772, 3800, and 3799, henceforth named LGMAI_St_08, LGMAI_St_11, and LGMAI_St_14, respectively. These strains were tested against *Streptococcus uberis* (*n* = 12), *Streptococcus porcinus* (*n* = 12), *Streptococcus pyogenes* (*n* = 19), *Streptococcus agalactiae* (*n* = 16), *Klebsiella pneumoniae* (*n* = 2), *Staphylococcus aureus* (*n* = 4), *Cellulomonas fimi,* and *Micrococcus* sp.

### 2.3. DNA Isolation and Illumina Hiseq Whole-Genome Sequencing

Strains LGMAI_St_08, LGMAI_St_11, and LGMAI_St_14 were selected for whole-genome sequencing due to their action spectra.

Genomic DNA was extracted using the phenol–chloroform method [28], with some modifications, and quantified by Qubit (Invitrogen, Waltham, MA, USA) fluorimetry. An amount of 5 μg/µL of gDNA was considered for the construction of paired-end sequencing libraries (2 × 150 bp) of 450 bp insert size, following the manufacturer’s protocol for the NEBNext^®^ Fast DNA Fragmentation and Library Preparation Kit (New England Biolabs Inc., Ipswich, MA, USA). Final library-quality analysis was performed via 2100 bioanalyzer (Agilent Technologies, Santa Clara, CA, USA) with read length gDNA size control using agarose gel electrophoresis. All strains were sequenced on the Illumina Hi-Seq 2500 platform, as recommended by the manufacturer.

### 2.4. Genome Assembly

The first step of the genome assembly process was to check the reads’ quality through FastQC [29] and remove the adapters with the Adapter Removal [30] software. The estimated best k-mers were selected by KmerStream [31], followed by the assembly using Edena [32] and SPAdes [33]. Then, the results were combined and the CD-HIT [34], package PSI-CD-HIT, was used to remove the redundant contigs, producing a final contigs file. Assembled genomes were then annotated using Prokka software [35].

### 2.5. Taxonomic Identification

We performed a taxonomic identification to properly classify the genomes before their submission to the GenBank database. This classification was carried out using the multilocus sequence analysis (MLSA) method. In addition to the 3 Streptococcus genomes sequenced in our study, another 36 Streptococcus species genomes were used for this analysis. *Lactococcus lactis* subsp. *lactis* and *Lactococcus lactis* subsp. *cremoris* were used as outgroups to root the phylogenetic tree (Table 2). The 16S rRNA, shikimate dehydrogenase (*aroE*), glucokinase (*gki*), phenylalanine-tRNA ligase alpha subunit (*pheS*), and *recA* genes were extracted from these genomes and used to construct the concatenated sequences for the phylogenetic analysis. The sequence alignment via Clustal W and phylogenetic tree inference were made in MEGA X software [36]. Phylogenetic tree inference was based on a maximum likelihood analysis with 1000 bootstraps, using the general time reversible model, with a gamma distribution and considering some nucleotide sites to be invariant.

*Streptococcus agalactiae* possesses a capsular polysaccharide, which is a virulence factor that grants the bacteria with immune system evasion and host cell invasion. There are, in total, ten different capsular types, and they are used in the species serotyping. The main serotypes identified in bovine mastitis infections are Ia, II, III, and V, whereas those associated with infections in humans are mainly Ia, Ib, II, III, and V [7]. Thus, we also performed a whole-genome sequence data-based identification of GBS capsular serotypes, as previously described by [37], to determine the isolates serotypes. For this analysis, LGMAI’s *Streptococcus agalactiae* subject genomes were blasted against query references for the ten different GBS capsular serotypes, as described in [37].

### 2.6. Assessments for Virulence Factors and Antibiotic Resistance

Bacteriocins are ribosomally synthesized antimicrobial peptides produced by bacteria. For instance, such compounds may grant bacteriocin-producing bacteria advantages in replication and settlement to different sites [38,39]. Thus, these substances have similar characteristics to bacterial virulence factors. Therefore, the ABRicate software [40] was used to analyze the presence of virulence factors in the *Streptococcus agalactiae* genomes that could be linked to the inhibition phenotypes observed. Furthermore, taking into consideration the rising levels of antimicrobial resistance identified in this species, the software was also used to look for the presence of antibiotic resistance genes in the strains’ genomes. ARG-ANNOT (Antibiotic Resistance Gene–ANNOTation) [41], CARD (Comprehensive Antibiotic Resistance Database) [42], NCBI AMRFinderPlus tool [43], and Resfinder [44] were used for the assessment of antibiotic resistance genes. The VFDB (Virulence Factors Database) [45] tool was used to identify virulence factors, and the GIPSy (Genomic Island Prediction Software) [46] software was used to predict pathogenicity and resistance islands. *Streptococcus thermophilus* JIM 8232 (assembly accession number GCA_000253395.1) was used as the non-pathogenic reference genome required for the GIPSy analysis.

### 2.7. Genome Mining for Antimicrobial Substances

The antibiotics and secondary metabolites analysis shell (antiSMASH) platform [47] was used to identify secondary metabolite biosynthesis gene clusters. The BAGEL4 [48] webserver was used to identify putative biosynthesis gene clusters of ribosomally synthesized and post-translationally modified peptides (RiPPs) and unmodified bacteriocins. Similarly, the BACTIBASE [49] web platform was used to identify bacteriocin genes and provide information about such peptides’ physicochemical and structural properties. For all these analyses, the *Streptococcus agalactiae* genome fasta files were used as input for the matching search with the databases. Undetected accessory genes were manually searched in the genomes’ annotation files.

Multiple sequence alignment for the bacteriocin zoocin A was performed using twelve zoocin A sequences obtained from the Uniprot database (Table 3).

### 2.8. Genome Comparison and Functional Annotation

The eggNOG-mapper tool [50] was used to analyze the *Streptococcus agalactiae* genomes and identify different clusters of orthologous groups (COGs). Genome comparisons were performed with the OrthoVenn2 web platform [51]. This platform annotates orthologous gene clusters and provides a comparison of multiple protein datasets via Venn diagrams. The BRIG software [52] was used to analyze the nucleotide identity among the LGMAI *Streptococcus agalactiae* genomes and 15 other *S. agalactiae* genomes obtained from NCBI (Table 4).

For the nucleotide identity comparison, the LGMAI_St_08 genome was used as the reference genome due to its larger genome size. Furthermore, predicted genomic islands were plotted onto the BRIG similarity plots to observe their conservation among the different *Streptococcus agalactiae* strains analyzed.

## 3. Results

### 3.1. Antimicrobial Activity Spectrum

The deferred growth inhibition assays demonstrated that 14 out of the 54 *Streptococcus agalactiae* strains inhibited at least 1 of the indicator strains. *Streptococcus agalactiae* I14 and *Streptococcus pyogenes* were the only indicator strains inhibited (Table 5).

These results indicate an inhibition pattern targeting *Streptococcus* species. Additional antagonism tests were performed against *Streptococcus uberis* (*n* = 12), *Streptococcus porcinus* (*n* = 12), *Streptococcus pyogenes* (*n* = 19), *Streptococcus agalactiae* (*n* = 16), *Klebsiella pneumoniae* (*n* = 2), *Staphylococcus aureus* (*n* = 4), *Cellulomonas fimi,* and *Micrococcus* sp. to test this hypothesis.

All four Streptococcus agalactiae producing strains selected for the additional tests inhibited at least 5 *Streptococcus porcinus*, 6 *Streptococcus pyogenes*, *15 Streptococcus agalactiae*, 4 *Streptococcus uberis*, *Cellulomonas fimi,* and the *Micrococcus* sp. Neither Klebsiella pneumoniae nor Staphylococcus aureus was inhibited by the Streptococcus agalactiae strains (Table 6). The four Streptococcus agalactiae strains did not show differences in terms of inhibition zones size, with all of them presenting mean values of 2 mm.

### 3.2. Genome Sequencing

The genomes belonging to the LGMAI_St_08, LGMAI_St_11, and LGMAI_St_14 strains were sequenced. The LGMAI_St_11 strains had the smallest genome size with 2,292,224 bp, while the LGMAI_St_08 strains had the largest genome size with 2,397,674 bp. Furthermore, GC content ranged from 35.6% to 35.7%. All three genomes presented a good number of predicted genes based on reference genomes. Complete detailed statistics on the resulting genomes are shown in Table 7.

Quality assessment for assembly completeness performed with the Busco software showed similar results for all three genomes, identifying all 124 universal single-copy orthologs for bacteria, among which 122 are complete, and 2 are fragmented.

### 3.3. Taxonomic Identification

**Figure 1:**
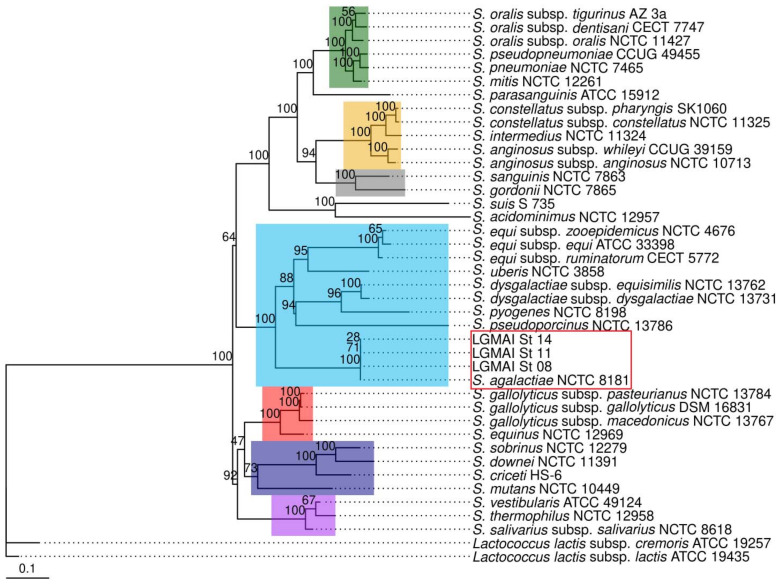
MLSA resultant phylogenetic tree, using the genes *aroE*, *gki*, *pheS*, *recA*, and the 16S rDNA, and inferred with the maximum likelihood method and the general time-reversible model. *Lactococcus lactis* subspp. *cremoris* and *lactis* were used as outgroups to root the tree. A rectangle with red borders highlights the bovine strains grouped with the reference *Streptococcus agalactiae* NCTC 8181. In colored rectangles are the streptococcal putative monophyletic groups found in the analysis (green = Mitis, yellow = Anginosus, gray = Sanguinis, light blue = Pyogenes, red = Bovis, dark blue = Mutans, and purple = Salivarius).

We inferred a phylogenetic tree to better understand the *Streptococcus* species’ relationships and confirm the *Streptococcus agalactiae* strains’ biochemical identification. The phylogenetic inference was performed via a multilocus sequence analysis using the maximum likelihood estimation method and the general time-reversible substitution model. This multilocus sequence analysis confirmed that the bovine strains belong to the *Streptococcus agalactiae* species, with strong bootstrap statistics supporting the branch. Moreover, the separation of the *Streptococcus* species in putative monophyletic groups, such as anginosus, bovis, mitis, mutans, pyogenes, salivarius, and sanguinis clusters was also detected. *Streptococcus parasanguinis* ATCC 15912, however, did not fall into the salivarius group (Figure 1).

**Figure 2:**
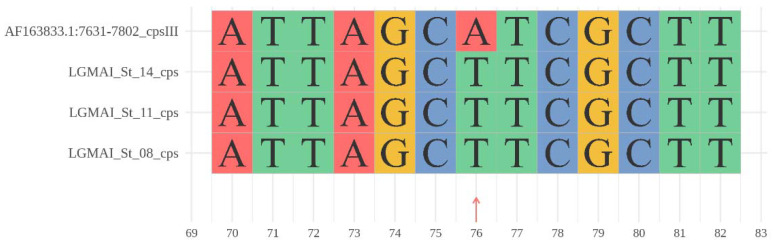
Whole-genome sequence data-based serotyping for *Streptococcus agalactiae.* Blast alignment among the three cps loci obtained from the LGMAI’s *Streptococcus agalactiae* genomes and the serotype III query sequence. Thirteen-nucleotide window extract showing the only mutation observed at position 76.

All three LGMAI’s *Streptococcus agalactiae* subject genomes matched the serotype III query sequence with 99% identity, superseding the analysis’s requirement. Only one mutation was observed at position 76 (Figure 2). Furthermore, none of the genomes additionally matched the serotype II query sequence, as sometimes observed, allowing for a clear identification as serotype III.

### 3.4. Antibiotic Resistance Genes and Virulence Factors

The assessments of antibiotic resistance genes performed with the CARD and Resfinder databases identified the genes *mprF* and *mreA,* with 99.49% and 99.79% identity, respectively, in all three genomes analyzed. The *mprF* gene encodes a phosphatidylglycerol lysyltransferase enzyme. The *mreA* gene encodes the protein responsible for riboflavin biosynthesis. No other antibiotic resistance genes were found either in the NCBI AMRFinderPlus tool or in the ARG-ANNOT databases.

The Virulence Factors Database identified 24 genes in the LGMAI_St_11 and LGMAI_St_14 genomes and 25 genes in the LGMAI_St_08 genome, representing important virulence factors such as the *cps* locus, the operon *cyl*, the hyaluronate lyase enzyme, and the CAMP factor. The *cfa*/*cfb* gene was detected exclusively in the LGMAI_St_08 genome (Table 8).

We used the GIPSy software to predict resistance and pathogenicity islands in the LGMAI’s *Streptococcus agalactiae* genomes. The analysis identified a total of a hundred and twenty putative genomic islands. After filtering the result for scores strong and normal, we registered eight genomic islands for the LGMAI_St_08 strain, seventeen for the LGMAI_St_11 strain, and fourteen for the LGMAI_St_14 strain (Table 9).

These predicted genomic islands were then plotted onto a circular genome map using the BRIG software to evaluate their conservation amongst different *Streptococcus agalactiae* strains.

### 3.5. Gene Clusters of Antimicrobial Peptides

The antiSMASH software analysis registered 14 clusters of secondary metabolites distributed among the three *Streptococcus agalactiae* genomes under investigation: LGMAI_St_08 (*n* = 4), LGMAI_St_11 (*n* = 5), and LGMAI_St_14 (*n* = 5). These gene clusters included type III polyketide synthase (*n* = 3), non-ribosomal peptide synthetase for equibactin (*n* = 5), lanthipeptide class V (*n* = 3), and aryl polyene (*n* = 3).

The BAGEL4 web server identified one area of interest in each genome. This area represents the gene cluster that regulates the production of the bacteriocin zoocin A. The zoocin A core peptide gene showed an identity of 58.76% with an e-value of $3\times 10^{-117}$. Similarly, the BACTIBASE database detected the antimicrobial peptide zoocin A in all three genomes analyzed. The zoocin A amino acid sequence showed an identity of 60% with an e-value of $3.44045\times 10^{-101}$.

**Figure 3:**
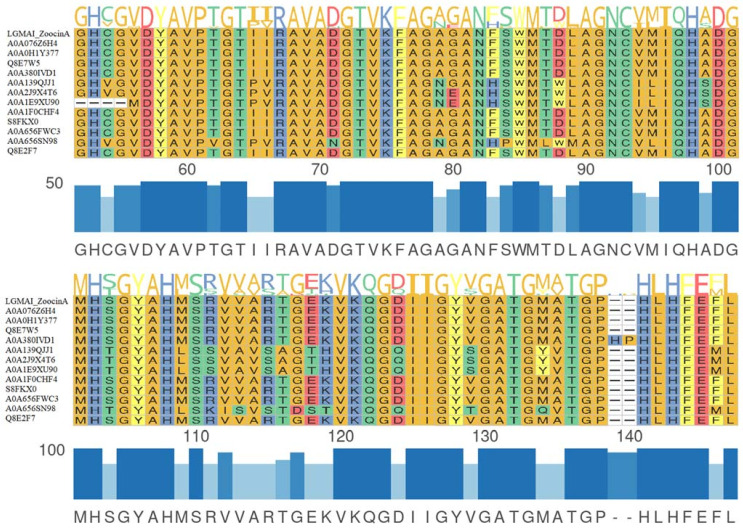
Multiple sequence alignment between the LGMAI’s putative zoocin A and 12 other zoocin A sequences obtained from the Uniprot database. Amino acid conservation can be observed in the M23 peptidase domain. Protein sequence logos are at the top of the representation, while multiple sequence alignments are in the middle, and the identity bars and consensus sequences are at the bottom.

**Figure 4:**
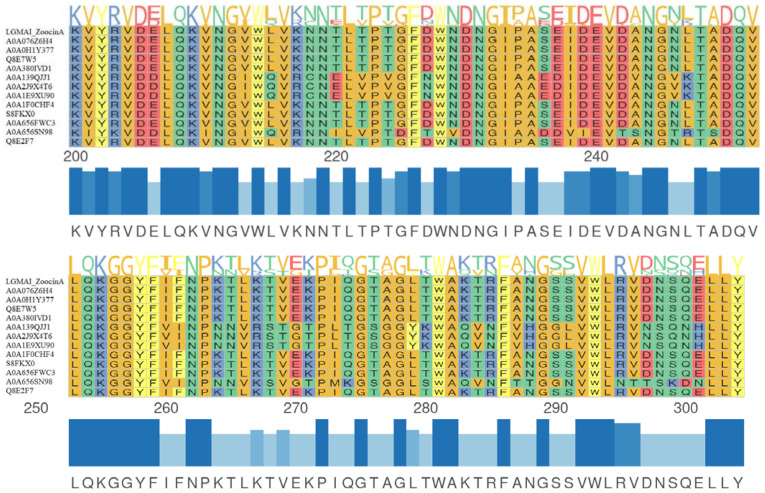
Multiple sequence alignment between the LGMAI’s putative zoocin A and 12 other zoocin A sequences obtained from the Uniprot database. Amino acid conservation can be observed in the target recognition domain. Protein sequence logos are at the top of the representation, while multiple sequence alignments are in the middle, and the identity bars and consensus sequences are at the bottom.

Protein sequence analysis on the InterPro database recognized a leader peptide sequence and two domains in the LGMAI’s putative zoocin A: the peptidase M23 catalytic domain and the lytic exoenzyme target recognition domain. Multiple sequence alignment between the LGMAI’s putative zoocin A and twelve other zoocin A sequences obtained from the Uniprot database showed amino acid conservation of the two domains (Figure 3 and Figure 4). Multiple sequence alignment revealed that the LGMAI’s putative zoocin A amino acid sequences were completely identical.

The zoocin A immunity factor (Zif) is a FemABX-like aminoacyl-transferase. The search for FemABX-like proteins in the genomes’ annotation files revealed the presence of FemB aminoacyl-transferases. LGMAI’s putative Zif sequences analysis on the InterPro database identified protein and superfamily regions, t-RNA binding arm, and coil (Table 10). These three sequences are identical and composed of 410 amino acid residues with 50% identity and 65% similarity to the Zif protein (UniProt entry P74894) from *Streptococcus equi* subsp. *zooepidemicus*.

### 3.6. Genome Comparison and Functional Annotation

The eggNOG-mapper tool registered 2169 orthologous groups for the LGMAI_St_08 strain, 2130 for the LGMAI_St_11 strain, and 2127 for the LGMAI_St_14 strain. Metabolism was the most abundant category among these groups (Table 11).

**Figure 5:**
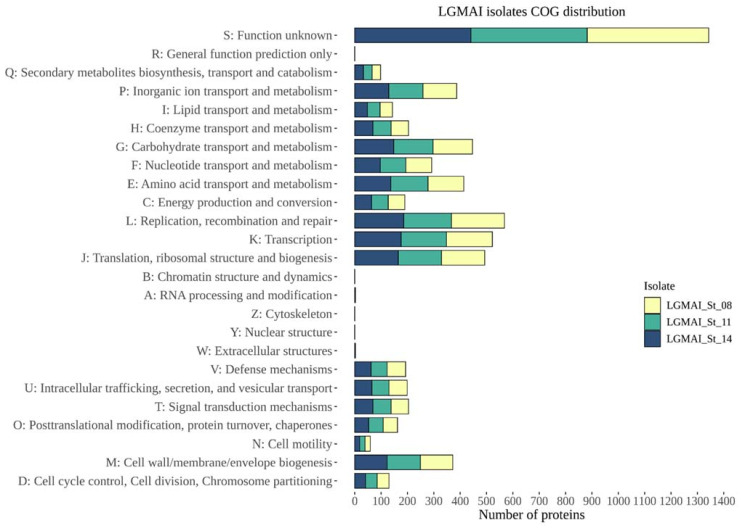
Stacked bar plot displaying the functional annotation of clusters of orthologous groups for the three LGMAI *Streptococcus agalactiae* strains. The number of proteins is on the *x*-axis, and COG categories are on the *y*-axis. Colors represent their respective LGMAI strain.

Overall, all three genomes showed similar results for the annotation of clusters of orthologous groups. Function unknown (S), replication, recombination, and repair (L), and transcription (K) were the most plentiful functional categories observed. Furthermore, we observed no clusters designated to the general function prediction only (R), chromatin structure and dynamics (B), cytoskeleton (Z), and nuclear structure (Y) functional categories (Figure 5).

**Figure 6:**
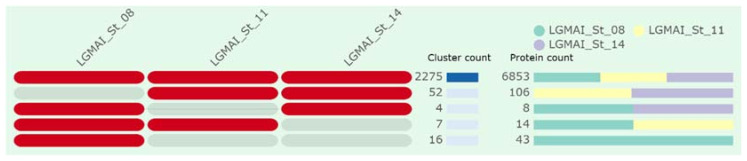
Cluster and protein counts identified by the genome comparison web server OrthoVenn2.

The genome comparison analysis showed that the three strains are very similar amongst themselves. A total of 2275 clusters are shared by all three strains, accounting for 6853 proteins (LGMAI_St_08: 2291 (33.43%), LGMAI_St_11: 2281 (33.28%), and LGMAI_St_14: 2281 (33.28%)). Pairwise comparisons of orthologous groups indicate that LGMAI_St_11 and LGMAI_St_14 are closer than the LGMAI_St_08 strain. They share 52 groups, accounting for 106 proteins (LGMAI_St_11: 52 (49.06%) and LGMAI_St_14: 54 (50.94%)). LGMAI_St_08 registered 16 exclusive orthologous groups composed of 43 proteins (Figure 6).

Molecular function clusters seem to be conserved, strictly represented in the overlap of the three strains. The overlap between strains LGMAI_St_08 and LGMAI_St_14 is the smallest, accounting for proteins related to extracellular region. Moreover, proteins responsible for cellular components, especially the categories for cell parts and cell membrane were also conserved. Most of the 16 orthologous groups found exclusively in LGMAI_St_08 belong to the biological processes category, with only one group for molecular function related to ion binding.

**Figure 7:**
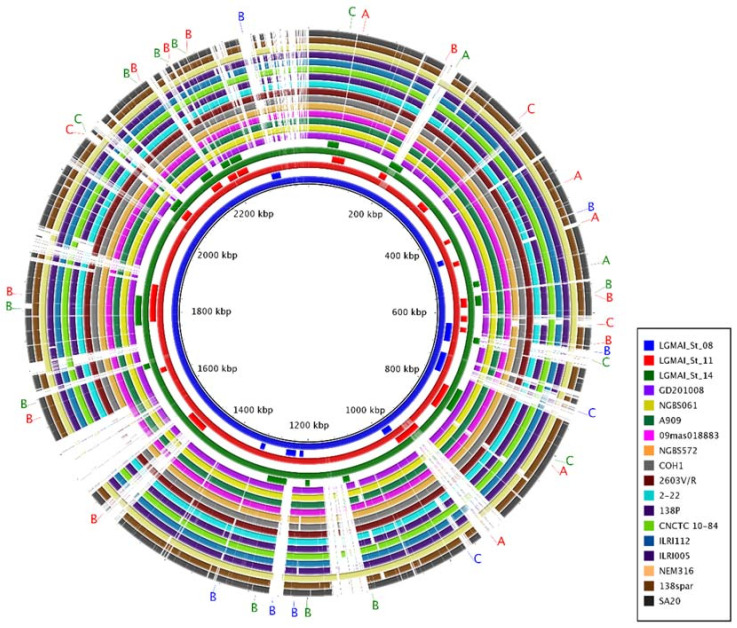
Circular genome map of the three LGMAI strains and 15 other well-represented *Streptococcus agalactiae* genomes obtained from NCBI. From the inside out, the genomes used in this analysis are in the legend order. The blue, red, and green inner circles represent the LGMAI strains. Outside each of the LGMAI’s genomes, predicted pathogenicity and resistance islands are plotted in the same colors of their corresponding strains. Letters indicate the genomic island types: A—resistance islands, B—pathogenicity islands, and C—miscellaneous islands. Letters’ colors match their corresponding genomes. This circular genome map shows nucleotide identity between the reference genome and the other genomes used in the analysis. Stronger colors register greater identities.

The circular genome map generated by the BRIG software shows that the three LGMAI strains share high similarities amongst themselves. In addition, they possess genomic regions that are not present in some of the other 15 genomes used for comparison in the analysis. Interestingly, some of the predicted pathogenicity and resistance islands overlap with parts of those regions (Figure 7).

## 4. Discussion

The deferred growth inhibition assays demonstrated that the LGMAI *Streptococcus agalactiae* strains produce bacteriocin-like substances. Additionally, the antagonism tests indicated an activity spectrum focused mainly on *Streptococcal* species, specifically *Streptococcus pyogenes*, *Streptococcus agalactiae*, *Streptococcus porcinus*, and *Streptococcus uberis*.

As frequently observed in studies of bacteriocins, these bacterial antimicrobial peptides are usually more prone to inhibiting closely related species [53]. In agreement with such expectations, we observed that the *Streptococcus agalactiae* bacteriocin-like substance showed an inhibition spectrum targeting mainly streptococcal species. From a clinical perspective, these results are interesting and promising because *Streptococcus* is a genus that comprises many relevant pathogenic bacteria [54].

*Streptococcus porcinus* accommodates similar beta-hemolytic streptococci that belong to Lancefield groups E, P, U, and V [55]. In humans, *Streptococcus porcinus* is responsible for urogenital tract infections in women. In pigs, it is associated with abortion events, lymph node and throat abscesses, pneumonia, and endocarditis. It is also related to cases of endometritis in swine after artificial insemination in farms. Antibiotic resistance is prevalent in this species, with some strains resistant to erythromycin, streptomycin, tetracycline, aminoglycosides, quinolones, macrolides, and others [56].

For instance, *Streptococcus uberis* and *Streptococcus agalactiae* are responsible for mastitis infections in animals [57,58], leading to both economic losses for farmers and health complications for the infected cattle. Antibiotic therapy is still one of the main ways to manage mastitis infections in cows. However, the issue of antimicrobial resistance asks for reductions and better usage of antibiotics, and that is also the case for antibiotics applied as preventive measures against infectious diseases in farms [59]. As a result, some alternative therapies to traditional antibiotics are considered, including phage therapy and phage enzymes, probiotics, and bacteriocins [60].

Since topical administration is more feasible for antimicrobial peptides [9], one could speculate topical application of the LGMAI strains’ substances to protect cattle udders and prevent infections that eventually result in mastitis and its subsequent implications. Nisin, for example, is a licensed food preservative used in over 50 countries [13]. Tests with nisin-containing sanitizer solutions and wipes have been carried out and showed satisfactory results on their efficacy in cleaning udder surfaces and preventing mastitis infections in cattle [60].

Not only do these species infect animals, but also humans. *Streptococcus agalactiae* (GBS) is a naturally occurring bacteria of the gastrointestinal and vaginal tracts that commonly produce no harm to their hosts. However, GBS infections can progress and result in bacteremia in adults and severe invasive GBS disease in newborns, this latter frequently fatal [58]. In particular, *Streptococcus pyogenes* is a pathogenic species infecting humans exclusively. Mainly found on the nasopharynx, these bacteria are responsible for causing mild to severe diseases, such as pharyngitis, scarlet fever, type II necrotizing fasciitis, and toxic shock syndrome [61]. Altogether, there are several streptococcal infections to be addressed in humans too. Additionally, CDC’s 2019 report on antimicrobial resistance classifies *Streptococcus pyogenes* and *Streptococcus agalactiae* as concerning threats. Their increasing resistance to clindamycin and erythromycin limits treatment options, requiring monitoring and preventive actions.

It is worth mentioning that the *Streptococcus agalactiae* strains were isolated from bovine mastitis. Therefore, their ability to inhibit other mastitis-causing streptococcal species, such as *Streptococcus uberis* and other *Streptococcus agalactiae* strains, possibly represents a way of competition for the cow’s udder site. This characteristic could be explored to address a search for alternative methods for dealing with mastitis infections in animals. Furthermore, future additional tests against other mastitis-causing bacteria should be considered.

The genomes presented GC content equivalent to the expected GC content for the *Streptococcus agalactiae* species and generated a good number of predicted genes/proteins. In addition, all three genomes passed the quality assessment for genome completeness with the Busco software, registering all the 124 universal single-copy orthologs, with only 2 of them fragmented.

The housekeeping genes *aroE*, *gki*, *pheS*, and *recA*, as well as the 16S rDNA, have already been used for taxonomic studies regarding the *Streptococcus* genus [62]. Here, we used these genes to generate concatenated sequences for the phylogenetic analysis of the LGMAI strains. For this, we performed a multilocus sequence analysis using the maximum likelihood method. The inferred phylogenetic tree allowed for the precise identification of the strains as *Streptococcus agalactiae,* with good bootstrap statistics support. *Streptococcus* species into the anginosus, bovis, mitis, mutans, pyogenes, salivarius, and sanguinis groups, as previously described [63,64], were also clearly retrieved. However, *Streptococcus parasanguinis* ATTC 15912 did not fall into the sanguinis group, requesting a more robust MLSA analysis with more orthologous proteins to better elucidate its group assignment.

Taxonomic studies for streptococcal bacteria using whole-genome sequencing (WGS) data are necessary because they enlighten much of the genus’ relationships, especially the relationships within the viridans streptococci, which are sometimes confusing [63]. Antibiotic resistance analysis revealed two genes, *mreA,* and *mprF*, in all three LGMAI strains’ genomes. The *mreA* gene encodes a riboflavin biosynthesis protein with flavokinase activity. This gene acts in a multidrug efflux pump manner and is associated with broad-spectrum resistance to many antimicrobial compounds, such as erythromycin, azithromycin, spiramycin, clindamycin, some cephalosporins, rifampicin, and others [65,66]. The *mprF* gene encodes a phosphatidylglycerol lysyltransferase that synthesizes lysylphosphatidylglycerol (LPG). LPG synthesis promotes resistance to various cationic antimicrobial peptides, such as defensins, cathelicidins, and bacteriocins, and thus also contributing to bacterial virulence [67,68]. The Centers for Disease Control and Prevention classify *Streptococcus agalactiae* as a concerning threat due to increasing resistance levels to clindamycin and erythromycin [69]. The *mreA* gene shows major significance to this extent since it is responsible for conferring resistance to erythromycin and clindamycin.

The virulence assessment analysis generated similar results in the three LGMAI genomes, accounting for important GBS virulence factors, such as the *cps* locus, *cyl* operon, and *hylB* gene. In addition, the *cfa*/*cfb* gene was found exclusively in the LGMAI_St_08 genome. The analysis identified 12 genes belonging to the cps locus—i.e., *cpsA*, *cpsB*, *cpsC*, *cpsD*, *cpsE*, *cpsF*, *cpsK*, *cpsL*, *neuA*, *neuB*, *neuC*, and *neuD*. All 12 genes mentioned are commonly shared by the 10 GBS serotypes [70]. This region encodes the gene products responsible for assembling of the GBS capsular polysaccharide (CPS), which is rich in sialic acid and encases *Streptococcus agalactiae*, facilitating bacteria immune evasion [71].

The analysis also identified 11 *cyl* operon genes—that is, *cylX*, *cylD*, *cylG*, *acpC*, *cylZ*, *cylA*, *cylB*, *cylF*, *cylI*, *cylJ*, and *cylK*. These genes are responsible for the synthesizing of the ornithine rhamnolipid pigment, also referred to as a hemolytic pigment due to its hemolytic activity [72]. This pigment contains a 12 double-bond polyene chain with potent cytotoxic activity and antioxidant properties, assisting infection and immune evasion processes [71]. The hemolytic pigment is associated with penetration of the human placenta and induction of loss of barrier function in human amniotic epithelial cells, promoting colonization of the feminine genital tract [72].

Cooperatively, *fbsB*, *hylB*, *cfa*/*cfb* genes, and pilus island PI-2b are vital during GBS infection in bovine mastitis [7]. The *hylB* gene encodes a hyaluronidase that acts as an endoglycosidase and cleaves glycosaminoglycan chains. Over-expression of the hyaluronidase has been demonstrated to confer hypervirulence for strains, even in those cases that lack the hemolytic pigment [71]. The degradation of the host hyaluronic acid releases its components, which bind to TLR2/TLR4 receptors and block their signaling pathways. Thus, this protein is crucial for immunosuppression during GBS infection [72].

The *cfa*/*cfb* gene was found exclusively in the LGMAI_St_08 genome. This gene produces the CAMP factor of *Streptococcus agalactiae*, which acts as a pore-forming toxin, ultimately lysing the host red blood cells [73]. The CAMP factor binds to the Fc site of IgM and IgG similarly to the *Staphylococcus aureus* protein A, which is responsible for immune evasion [74]. Both *hylB* and *cfa*/*cfb* genes cause tissue damage and are essential to GBS infection in cattle [7]. The identified virulence genes comprise the main virulence factors of *Streptococcus agalactiae*, but none of them show any correlation with the inhibition phenotype observed in the deferred growth assays.

The GIPSy software predicted eight genomic islands for the LGMAI_St_08 strain, seventeen for the LGMAI_St_11 strain, and fourteen for the LGMAI_St_14 strain. Comparative analysis, via mean nucleotide identity, revealed that most of the identified genomic islands represent conserved areas among *Streptococcus agalactiae*. Some of the LGMAI strains’ putative islands, however, are not present in the genomes used for comparison.

Despite the vital role of bacteriophage sequences in *Streptococcus agalactiae* strains’ variability and evolution [75], we could not recover any intact phage sequences from any of the three LGMAI strains’ genomes. Nonetheless, two questionable phage sequences were identified in the LGMAI_St_08 genome. Otherwise, only incomplete bacteriophage sequences were registered for LGMAI_St_11 and LGMAI_St_14 strains.

The antiSMASH analysis of antibiotics and secondary metabolites identified putative sites for producing type III polyketide synthases, aryl polyenes, and class V lanthipeptide. Polyketides are natural products that possess biologically active properties, such as antineoplastic, cholesterol-reducing, and antimicrobial [76]. On the other hand, aryl polyenes represent bacterial pigments similar to the carotenoids that protect bacteria against reactive oxygen species [77]. Lastly, lanthipeptides are ribosomally synthesized peptides that contain the lanthionine amino acid on their structure. These peptides may have several biologically active properties, including antimicrobial. Lanthipeptides with antimicrobial activity are called lantibiotics [78]. However, no core peptide was identified for these sites.

The antiSMASH webserver also recognized one biosynthetic cluster for the nonribosomal peptide-synthetase (NRPS) equibactin. The equibactin system was first observed in *Streptococcus equi* subsp. *equi* and is associated with iron uptake [79]. It is worth mentioning that iron acquisition is a vital process for pathogenic bacteria because available iron is usually scarce in mammalian cells. Equibactin is analogous to the yersiniabactin system from *Yersinia* sp. Therefore, equibactin may play a role in *Streptococcus agalactiae* virulence [80].

The BAGEL4 database recognized the zoocin A core peptide, showing 58.76% identity, in all three LGMAI genomes. The bacteriocin zoocin A is a lytic exoenzyme first identified in *Streptococcus equi* subsp. *zooepidemicus* 4881. This bacteriocin is an endopeptidase responsible for hydrolyzing the peptidoglycan structure of susceptible organisms [81]. Moreover, the zoocin A activity spectrum has been shown to strongly target *Streptococcus* spp., as also observed in the antagonism assays performed in this study [82]. In the same fashion, the BACTIBASE database analysis also showed correspondence to the zoocin A core peptide from *Streptococcus equi* subsp. *zooepidemicus*, with 60% identity and 66% similarity. This antimicrobial peptide contains two domains: an N-terminal catalytic domain (peptidase M23) and a C-terminal target recognition domain (TRD) [83]. Such domains were successfully identified via InterPro analysis of the zoocin A protein sequences.

It is noteworthy that the BACTIBASE database does not account for zoocin A inhibitory activity against *Micrococcus luteus* and *Streptococcus uberis*. The LGMAI bacteriocin-like substances studied herein did inhibit *Micrococcus* sp. and *Streptococcus uberis* indicator strains, suggesting sequence variation and evolution for the putative zoocin A antimicrobial substance identified in the LGMAI strains.

Apart from the zoocin A core peptide, the immunity factor, also referred to as Zif, protects the producing bacteria and is a vital part of this bacteriocin activity scheme. The Zif factor is a FemABX-like protein that blocks binding to the catalytic and recognition domains via adding an extra l-alanine during the peptidoglycan biosynthesis [84]. The Prokka software annotated aminoacyl transferases FemB in all three LGMAI genomes. These sequences were submitted to families and domains analysis on the InterPro database, which resulted in identifying the FemABX peptidyl transferase family of proteins matching positions.

The analysis with the eggNOG-mapper tool showed a similar distribution of orthologous groups among the three LGMAI strains. Nevertheless, the LGMAI_St_11 and LGMAI_St_14 genomes share more orthologous groups between each other than they do with the LGMAI_St_08 genome.

Comparative genomics with the OrthoVenn2 webserver corroborated the previous observations. The three genomes share many orthologous groups, with a slightly more significant similarity between the LGMAI_St_11 and LGMAI_St_14 genomes. In addition, proteins for molecular function and cellular components are more conserved, while proteins for metabolic processes are more variable than those mentioned above.

Furthermore, it is interesting to mention that the LGMAI_St_08 genome registered 16 exclusive orthologous functional groups. Most of these groups are related to biological processes, which may be associated with adaptations to the LGMAI_St_08 environment.

## 5. Conclusions

The deferred growth inhibition assays showed that the LGMAI *Streptococcus agalactiae* strains produce antimicrobial substances with an activity spectrum targeting streptococcal species—specifically, *Streptococcus pyogenes*, *Streptococcus agalactiae*, *Streptococcus uberis,* and *Streptococcus porcinus*. The LGMAI strains also inhibited *Micrococcus* sp. and *Cellulomonas fimi*. The multilocus sequence analysis and the CDC’s WGS GBS serotyping determined that the three strains are *Streptococcus agalactiae* serotype III.

Additionally, based on functional annotations and comparative genomics, the LGMAI_St_11 and LGMAI_St_14 strains share more similarities between them. These results are in agreement with their closer sampling geographic locality. Moreover, the three strains show high conservation of genes related to molecular function and cellular components.

Furthermore, assessments for antibiotic resistance genes identified the *mreA* gene in the three LGMAI genomes. Such a gene encodes a flavokinase and is related to resistance to erythromycin. Because of elevated resistance to erythromycin, *Streptococcus agalactiae* is already considered a concerning threat. Therefore, this finding contributes to the monitoring of antimicrobial resistance in GBS. Moreover, assessments for virulence factors recovered almost all of the main GBS virulence factors. The FbsA, FbsB, Lmb adherence proteins, pilus islands, and the *cba* gene remained unidentified.

Finally, putative sites were found for antibiotics and secondary metabolites with diverse biological activities, specifically antimicrobial, antioxidant, and iron uptake. Furthermore, the gene for the bacteriocin zoocin A, which targets streptococcal species, was also detected. Altogether, the antibiosis results and the *zooA* gene endorse the production of an antimicrobial substance by the LGMAI *Streptococcus agalactiae* strains. This substance targets pathogenic streptococci—notably, *Streptococcus pyogenes*, *Streptococcus agalactiae*, *Streptococcus uberis*, and *Streptococcus porcinus*.

## Figures and Tables

**Table 1:** *Streptococcus agalactiae* strains tested for antimicrobial substances production.

Strains	Source
3725, 3726, 3727, 3728, 3729, 3730, 3731, 3739, 3740, 3741, 3742, 3743, 3744, 3745, 3751, 3752	Farm 1 *—Minas Gerais, Brazil
3770, 3771, 3772, 3773, 3774, 3775, 3776	Farm 2 *—São Paulo, Brazil
3797, 3798, 3799, 3800, 3801, 3802, 3804	Farm 3 *—Minas Gerais, Brazil
3891, 3892, 3893, 3894, 3895, 3896, 3897, 3898, 3899	Farm 4 *—Minas Gerais, Brazil
129, 194, 835, 962, 1312, 1315, 3191, 3327, 3333, 3339, 3340, 3345, 3370, 3834, 3835	NA

NA: Not available. * The publication of names and precise geographical localizations were not allowed.

**Table 2:** Genomes used in the phylogenetic analysis and their respective NCBI accession numbers.

Genomes	Accession Numbers
*Streptococcus acidominimus* NCTC 12957	GCA_900459045.1
*Streptococcus agalactiae* NCTC 8181	GCA_900458965.1
*Streptococcus anginosus* subsp. *anginosus* NCTC 10713	GCA_900636475.1
*Streptococcus anginosus* subsp. *whileyi* CCUG 39159	GCA_000257765.1
*Streptococcus constellatus* subsp. *constellatus* NCTC 11325	GCA_900459125.1
*Streptococcus constellatus* subsp. *pharyngis* SK 1060	GCA_000223295.2
*Streptococcus criceti* HS 6	GCA_000187975.3
*Streptococcus downei* NCTC 11391	GCA_900459175.1
*Streptococcus dysgalactiae* subsp. *dysgalactiae* NCTC 13731	GCA_900459225.1
*Streptococcus dysgalactiae* subsp. *equisimilis* NCTC 13762	GCA_900459095.1
*Streptococcus equi* subsp. *equi* ATCC 33398	GCA_900156215.1
*Streptococcus equi* subsp. *ruminatorum* CECT 5772	GCA_000706805.1
*Streptococcus equi* subsp. *zooepidemicus* NCTC 4676	GCA_900459475.1
*Streptococcus equinus* NCTC 12969	GCA_900459295.1
*Streptococcus gallolyticus* subsp. *gallolyticus* DSM 16831	GCA_002000985.1
*Streptococcus gallolyticus* subsp. *macedonicus* NCTC 13767	GCA_900459545.1
*Streptococcus gallolyticus* subsp. *pasteurianus* NCTC 13784	GCA_900478025.1
*Streptococcus gordonii* NCTC 7865	GCA_900475015.1
*Streptococcus intermedius* NCTC 11324	GCA_900475975.1
*Streptococcus mitis* NCTC 12261	GCA_000148585.3
*Streptococcus mutans* NCTC 10449	GCA_900475095.1
*Streptococcus oralis* subsp. *dentisani* CECT 7747	GCA_000382825.1
*Streptococcus oralis* subsp. *oralis* NCTC 11427	GCA_900637025.1
*Streptococcus oralis* subsp. *tigurinus* AZ 3a	GCA_000344275.1
*Streptococcus parasanguinis* ATCC 15912	GCA_000164675.2
*Streptococcus pneumoniae* NCTC 7465	GCA_001457635.1
*Streptococcus pseudopneumoniae* CCUG 49455	GCA_002087075.1
*Streptococcus pseudoporcinus* NCTC 13786	GCA_900637075.1
*Streptococcus pyogenes* NCTC 8198	GCA_002055535.1
*Streptococcus salivarius* subsp. *salivarius* NCTC 8618	GCA_900636435.1
*Streptococcus sanguinis* NCTC 7863	GCA_900475505.1
*Streptococcus sobrinus* NCTC 12279	GCA_900475395.1
*Streptococcus suis* S735	GCA_000294495.1
*Streptococcus thermophilus* NCTC 12958	GCA_900474985.1
*Streptococcus uberis* NCTC 3858	GCA_900475595.1
*Streptococcus vestibularis* ATCC 49124	GCA_000188295.1
*Lactococcus lactis* subsp. *cremoris* ATCC 19257 (outgroup)	GCA_004354515.1
*Lactococcus lactis* subsp. *lactis* ATCC 19435 (outgroup)	GCA_900099625.1

**Table 3:** UniProt entries for the protein sequences used in the multiple sequence alignment against the LGMAI’s putative zoocin A.

UniProt Entry	Organism	Protein	Size
A0A076Z6H4	*Streptococcus agalactiae*	Peptidase (M23/M37)	299
A0A0H1Y377	*Streptococcus agalactiae*	Peptidase (M23/M37)	299
Q8E7W5	*Streptococcus agalactiae* serotype III (NEM316)	Uncharacterized	299
A0A380IVD1	*Streptococcus agalactiae*	Peptidase (M23/M37)	301
A0A139QJJ1	*Streptococcus constellatus*	Peptidase (M23/M37)	285
A0A2J9X4T6	*Streptococcus* sp. FDAARGOS_146	Zoocin A	255
A0A1E9XU90	*Streptococcus* sp. HMSC034B05	Zoocin A	238
A0A1F0CHF4	*Streptococcus* sp. HMSC069D09	Zoocin A	299
S8FKX0	*Streptococcus agalactiae* FSL S3-277	Zoocin A	299
A0A656FWC3	*Streptococcus agalactiae* (ATCC 13813/DSM 2134/JCM 5671/NCIMB 701348/NCTC 8181)	Peptidase (M23/M37)	299
A0A656SN98	*Streptococcus equi* subsp. *zooepidemicus* SzAM60	Peptidase (M24/M37)	285
Q8E2F7	*Streptococcus agalactiae* serotype V (ATCC BAA-611/2603 V/R)	Peptidase (M23/M37)	299

**Table 4:** Complete *Streptococcus agalactiae* genomes used in the nucleotide identity comparison performed with the BRIG software.

Genome	NCBI Accession Number	Size (bp)
*Streptococcus agalactiae* 2-22	GCA_000967445.1	1.838.867
*Streptococcus agalactiae* 09mas018883	GCF_000427035.1	2.138.694
*Streptococcus agalactiae* 138P	GCA_000599965.1	1.838.701
*Streptococcus agalactiae* 138spar	GCA_000636115.1	1.838.126
*Streptococcus agalactiae* 2603V/R	GCF_000007265.1	2.160.267
*Streptococcus agalactiae* A909	GCF_000012705.1	2.127.839
*Streptococcus agalactiae* CNCTC 10/84	GCF_000782855.1	2.013.842
*Streptococcus agalactiae* COH1	GCF_000689235.1	2.065.074
*Streptococcus agalactiae* GD201008-001	GCF_000299135.1	2.063.112
*Streptococcus agalactiae* ILRI005	GCF_000427075.1	2.109.759
*Streptococcus agalactiae* ILRI112	GCA_000427055.1	2.029.198
*Streptococcus agalactiae* NEM316	GCF_000196055.1	2.211.485
*Streptococcus agalactiae* NGBS061	GCF_000730215.1	2.221.207
*Streptococcus agalactiae* NGBS572	GCF_000730255.1	2.061.426
*Streptococcus agalactiae* SA20	GCA_000302475.3	1.841.952

Source: NCBI.

**Table 5:** Deferred growth inhibition assays results. Only displayed in the table are the *Streptococcus agalactiae* strains that inhibited at least one of the indicator strains. (+) indicates clear inhibition zones of at least 2 mm in size; (−) indicates absence of inhibition.

*Streptococcus agalactiae* Strains	Indicator Strains					
	*Cellulomonas fimi* NCTC 7547	*Listeria innocua*	*Staphylococcus aureus*	*Streptococcus agalactiae* I14	*Streptococcus pyogenes*	*Lactococcus lactis*
3770	−	−	−	+	−	−
3771	−	−	−	+	+	−
3772	−	−	−	+	+	−
3773	−	−	−	+	+	−
3774	−	−	−	+	+	−
3775	−	−	−	+	+	−
3776	−	−	−	+	+	−
3797	−	−	−	+	+	−
3798	−	−	−	+	+	−
3799	−	−	−	+	+	−
3800	−	−	−	+	+	−
3801	−	−	−	+	+	−
3802	−	−	−	+	+	−
3804	−	−	−	+	+	−

**Table 6:** Additional deferred growth inhibition assays results. Additional tests were performed with strains LGMAI_St_08 (3772), LGMAI_St_11 (3800), and LGMAI_St_14 (3799). (+) indicates clear inhibition zones of at least 2 mm in size; (−) indicates absence of inhibition.

Indicator Strains		*Streptococcus agalactiae* Strains		
Species	Strains	LGMAI_St_08	LGMAI_St_11	LGMAI_St_14
*Streptococcus agalactiae*	3725	+	+	+
	3726	+	+	+
	3727	+	+	+
	3728	+	+	+
	3729	+	+	+
	3730	+	+	+
	3731	+	+	+
	3739	+	+	+
	3740	+	+	+
	3741	+	+	+
	3742	+	+	+
	3743	+	+	+
	3744	−	−	−
	3745	+	+	+
	3751	+	+	+
	3752	+	+	+
*Streptococcus porcinus*	628	−	−	−
	662	+	+	+
	790	−	−	−
	857	+	+	+
	1058	+	+	+
	1124	+	−	−
	1217	+	+	+
	1451	−	−	−
	2378	−	−	−
	3123	+	+	+
	3176	−	−	−
	3658	−	−	−
*Streptococcus pyogenes*	1465	−	−	−
	1471	−	−	−
	1474	−	−	−
	1996	−	−	−
	2009	−	−	−
	2586	+	+	+
	2587	+	+	+
	2588	+	+	+
	2590	−	+	+
	2591	+	−	+
	2593	−	−	−
	2606	−	−	−
	2608	−	−	−
	2612	−	−	−
	2615	−	−	−
	2617	−	−	−
	2618	+	+	+
	2620	−	−	−
	2625	+	−	−
*Streptococcus uberis*	602	+	−	−
	752	−	−	−
	959	−	−	−
	2825	+	−	−
	3355	+	+	−
	3351	+	+	+
	3354	+	+	+
	3431	−	−	−
	3485	+	−	+
	3670	+	+	+
	3671	+	+	+
	3724	−	−	−
*Cellulomonas fimi*		+	+	+
*Micrococcus* sp.		+	+	+
*Klebsiella pneumoniae*	ATCC 13883	−	−	−
	KPC	−	−	−
*Staphylococcus aureus*	ATCC 6538	−	−	−
	ATCC 29213	−	−	−
	ATCC 25923	−	−	−
	ATCC 33591	−	−	−

**Table 7:** Genome statistics obtained from assemblers and side analyses.

Parameter	Strains		
	LGMAI_St_08	LGMAI_St_11	LGMAI_St_14
GenBank accession number	JAIWPA000000000	JAIWPB000000000	JAIWPC000000000
BioSample accession number	SAMN17072134	SAMN17072135	SAMN17072136
BioProject accession number	PRJNA637496		
Total genome size	2,397,674	2,292,224	2,289,478
Number of contigs	156	334	292
N50	33,723	46,037	45,516
L50	25	16	18
GC content (%)	35.7%	35.6%	35.6%
Number of genes	2499	2502	2450
Number of CDS	2434	2452	2400
Number of coding genes	2334	2361	2310
rRNA	10	6	5
tRNA	52	41	42
Pseudo Genes	100	91	90

**Table 8:** Virulence genes identified by the Virulence Factor Database. The *cpsE* gene had a coverage of 99.35%. All other genes showed 100% coverage. Percentages shown in the table represent gene identities per strain.

Genes		Strains		
		LGMAI_St_08	LGMAI_St_11	LGMAI_St_14
Locus *cps*	*cpsA*	100%	100%	100%
	*cpsB*	100%	100%	100%
	*cpsC*	100%	100%	100%
	*cpsD*	100%	100%	100%
	*cpsE*	99.35%	99.35%	99.35%
	*cpsF*	100%	100%	100%
	*cpsK*	100%	100%	100%
	*cpsL*	100%	100%	100%
	*neuA*	100%	100%	100%
	*neuB*	100%	100%	100%
	*neuC*	100%	100%	100%
	*neuD*	100%	100%	100%
Operon *cyl*	*cylX*	100%	100%	100%
	*cylD*	100%	100%	100%
	*cylG*	100%	100%	100%
	*acpC*	100%	100%	100%
	*cylZ*	100%	100%	100%
	*cylA*	95.27%	100%	100%
	*cylB*	100%	100%	100%
	*cylF*	100%	100%	100%
	*cylI*	100%	100%	100%
	*cylJ*	100%	100%	100%
	*cylK*	100%	100%	100%
*hylB*		100%	100%	100%
*cfa*/*cfb*		100%	NA	NA

**Table 9:** Extract from complete genomic island prediction results depicting only the predicted genomic islands with scores strong and normal. Res: resistance island; Pat: pathogenicity island; Misc: miscellaneous island.

Strain	Genomic Island	GC Deviation	Codon Usage Deviation	Specific Proteins	Hypothetical Proteins	Position	Score
LGMAI_St_08	Pat 1	50%	25%	41%	66%	456,093–470,295	Normal
	Pat 2	26%	19%	46%	61%	626,248–675,463	Normal
	Pat 3	13%	0%	100%	53%	1,212,789–1,222,960	Normal
	Pat 4	17%	70%	52%	58%	1,232,986–1,259,538	Normal
	Pat 5	23%	0%	76%	46%	1,318,556–1,330,577	Normal
	Pat 6	16%	6%	80%	96%	2,297,372–2,321,939	Normal
	Misc 1	25%	39%	20%	70%	706,904–759,427	Normal
	Misc 2	32%	56%	24%	48%	961,688–986,574	Normal
LGMAI_St_11	Res 1	20%	29%	8%	58%	58,075–88,763	Normal
	Res 2	14%	0%	42%	57%	414,714–424,358	Normal
	Res 3	0%	21%	21%	71%	471,775–483,184	Normal
	Res 4	6%	43%	19%	67%	782,301–843,622	Normal
	Res 5	20%	57%	11%	70%	905,416–968,419	Normal
	Pat 1	6%	6%	43%	68%	181,057–198,487	Normal
	Pat 2	4%	0%	100%	26%	564,594–586,997	Normal
	Pat 3	0%	90%	45%	72%	634,882–646,520	Strong
	Pat 4	18%	23%	46%	55%	1,477,345–1,527,804	Normal
	Pat 5	15%	0%	76%	46%	1,650,399–1,662,421	Normal
	Pat 6	1%	33%	43%	78%	1,776,966–1,868,151	Normal
	Pat 7	23%	53%	42%	69%	2,144,963–2,170,746	Normal
	Pat 8	14%	35%	67%	85%	2,192,667–2,214,594	Normal
	Pat 9	7%	0%	40%	88%	2,217,596–2,244,976	Normal
	Misc 1	6%	100%	20%	53%	302,365–327,227	Strong
	Misc 2	26%	86%	26%	53%	607,341–620,962	Strong
	Misc 3	20%	45%	12%	58%	2,042,812–2,067,825	Normal
LGMAI_St_14	Res 1	23%	29%	8%	58%	190,826–221,514	Normal
	Res 2	0%	33%	26%	66%	528,604–541,172	Normal
	Pat 1	4%	0%	100%	26%	561,516–583,919	Normal
	Pat 2	21%	0%	78%	42%	1,105,918–1,120,919	Normal
	Pat 3	41%	50%	41%	66%	1,195,840–1,205,898	Normal
	Pat 4	25%	29%	51%	65%	1,247,996–1,291,657	Normal
	Pat 5	15%	0%	76%	46%	1,670,544–1,682,566	Normal
	Pat 6	0%	11%	52%	71%	1,770,456–1,836,354	Normal
	Pat 7	32%	21%	46%	67%	2,139,499–2,166,899	Normal
	Pat 8	15%	33%	66%	84%	2,193,909–2,213,031	Normal
	Pat 9	11%	0%	46%	92%	2,216,033–2,242,629	Normal
	Misc 1	6%	100%	20%	53%	43,216–68,078	Strong
	Misc 2	20%	93%	20%	60%	655,073–668,583	Strong
	Misc 3	15%	51%	23%	71%	778,157–830,840	Normal
	Misc 4	25%	42%	10%	53%	2,044,364–2,073,403	Normal

**Table 10:** Protein family regions identified by the InterPro LGMAI’s putative Zif sequences analysis.

Region	Database	Accession Number	Position	e-Value
FemABX peptidyl transferase	Pfam	PF02388	6–406	$1.1\times 10^{-147}$
Acyl-CoA N-acyltransferase	Superfamily	SSF55729	1–159	$5.3\times 10^{-43}$
Acyl-CoA N-acyltransferase			163–403	$2.7\times 10^{-28}$
Class I and II aminoacyl-tRNA synthetase		SSF46589	239–303	$7.3\times 10^{-7}$
(tRNA-binding arm)	Coils	Coil	243–263	NA

NA: Not applicable.

**Table 11:** Distribution of the orthologous groups identified by the eggNOG-mapper tool among the 4 major COG categories.

Category	Strains		
	LGMAI_St_08	LGMAI_St_11	LGMAI_St_14
Cellular processes and signaling	448	441	433
Information storage and processing	540	518	528
Metabolism	720	730	725
Poorly characterized	461	441	441

## Data Availability

The genomes’ sequencing project has been deposited in the NCBI under BioProject accession number PRJNA637496. The *Streptococcus agalactiae* LGMAI_St_08, LGMAI_St_11, and LGMAI_St_14 genomes have been deposited in the NCBI under the accession numbers JAIWPA000000000, JAIWPB000000000, and JAIWPC000000000, respectively.
